# Prediction of Poor Outcome after Successful Thrombectomy in Patients with Severe Acute Ischemic Stroke: A Pilot Retrospective Study

**DOI:** 10.3390/neurolint15010015

**Published:** 2023-02-03

**Authors:** Burak B. Ozkara, Mert Karabacak, Apoorva Kotha, Alperen Aslan, Omar Hamam, Namratha Edpuganti, Meisam Hoseinyazdi, Richard Wang, Brian C. Cristiano, Vivek S. Yedavalli

**Affiliations:** 1 Department of Neuroradiology, MD Anderson Cancer Center, 1400 Pressler Street, Houston, TX 77030, USA; 2 Department of Neurosurgery, Mount Sinai Health System, 1468 Madison Avenue, New York, NY 10029, USA; 3 Russell H. Morgan Department of Radiology and Radiological Science, Johns Hopkins Hospital, 600 N Wolfe Street, Baltimore, MD 21287, USA; 4 Department of Interventional Neuroradiology, Johns Hopkins Hospital, 600 N Wolfe Street, Baltimore, MD 21287, USA

**Keywords:** acute ischemic stroke, mechanical thrombectomy, prognosis, platelet count

## Abstract

Several baseline hematologic and metabolic laboratory parameters have been linked to acute ischemic stroke (AIS) clinical outcomes in patients who successfully recanalized. However, no study has directly investigated these relationships within the severe stroke subgroup. The goal of this study is to identify potential predictive clinical, lab, and radiographic biomarkers in patients who present with severe AIS due to large vessel occlusion and have been successfully treated with mechanical thrombectomy. This single-center, retrospective study included patients who experienced AIS secondary to large vessel occlusion with an initial NIHSS score ≥ 21 and were recanalized successfully with mechanical thrombectomy. Retrospectively, demographic, clinical, and radiologic data from electronic medical records were extracted, and laboratory baseline parameters were obtained from emergency department records. The clinical outcome was defined as the modified Rankin Scale (mRS) score at 90 days, which was dichotomized into favorable functional outcome (mRS 0–3) or unfavorable functional outcome (mRS 4–6). Multivariate logistic regression was used to build predictive models. A total of 53 patients were included. There were 26 patients in the favorable outcome group and 27 in the unfavorable outcome group. Age and platelet count (PC) were found to be predictors of unfavorable outcomes in the multivariate logistic regression analysis. The areas under the receiver operating characteristic (ROC) curve of models 1 (age only model), 2 (PC only model), and 3 (age and PC model) were 0.71, 0.68, and 0.79, respectively. This is the first study to reveal that elevated PC is an independent predictor of unfavorable outcomes in this specialized group.

## 1. Introduction

Acute ischemic stroke (AIS) is a significant worldwide cause of morbidity, with 12.2 million new cases per year [1]. Large vessel occlusion (LVO) is the underlying cause in approximately 30% of patients with AIS [2]. Approximately 80% of LVO-AIS cases occur in the anterior circulation [3].

Mechanical thrombectomy (MT) has become the standard of care in treating patients presenting with AIS secondary to LVO, where recent landmark trials have shown improved functional outcomes in these patients for up to 24 h after symptom onset [4,5]. Nevertheless, patients who present with severe AIS (defined as an admission National Institutes of Health Stroke Scale (NIHSS) score ≥ 21) secondary to LVO have unfavorable outcomes, even with successful MT. Even though successful revascularization increases the probability of favorable clinical results following treatment in this subgroup, not all patients who undergo successful revascularization experience a favorable clinical outcome. Advanced age, high presentation NIHSS score, non-recanalization (modified Thrombolysis in Cerebral Infarction (mTICI) ≤ 2a), post-procedural reperfusion hemorrhage, presenting ischemic core volume, baseline computed tomography (CT) perfusion profile, and index occlusion of the internal carotid artery (ICA) have all been established as predictors of poor outcome after MT in multiple large logistic regression analyses [6,7,8,9,10].

There is a growing interest in more prognostic indicators for reperfusion stroke therapies that can be used early on, including clinical, imaging, and laboratory parameters [11,12,13]. Specifically, various baseline hematologic and metabolic lab parameters have been associated with clinical outcomes in AIS. In addition, there is continuing research on prediction models that might aid clinical decision-making using features believed to be linked with certain outcomes. A binary outcome prediction model based on logistic regression can improve clinical decision-making. However, no study to date has directly investigated these relationships within the severe stroke subgroup where the prognosis is relatively poor [14].

Therefore, the aim of this study is to determine potential predictive clinical, lab, and radiographic biomarkers in patients who present with an NIHSS score ≥ 21 secondary to LVO and who were successfully treated with MT. To the best of our knowledge, this would be the first study to explore these relationships within this specific subgroup.

## 2. Material and Methods

### 2.1. Patient Selection

This single-center, retrospective cohort study included patients with AIS with LVO in the anterior circulation who were admitted between 1 September 2019 and 1 April 2021 to Johns Hopkins Hospital. LVO was defined as distal intracranial ICA, M1, and proximal M2 segments of the middle cerebral artery [15]. AIS was clinically diagnosed and confirmed with brain CT or magnetic resonance imaging (MRI). The inclusion criteria were as follows: (1) admitted to Johns Hopkins Hospital within 24 h of symptoms of stroke onset; (2) ≥18 years of age; (3) initial non-contrast brain CT scan data to exclude the intracranial hemorrhage; (4) ≥21 NIHSS score at admission; and (5) successful recanalization with MT. Patients with missing data, intracranial hemorrhage, aged less than 18 years, unsuccessful recanalization with mTICI less than 2b or a failed attempt of MT, and those with a discharge diagnosis of the transient ischemic attack were excluded. Successful recanalization was defined as an mTICI score of 2b or higher as determined by the performing neurointerventionalist [16]. The study received Institutional Review Board approval at Johns Hopkins Hospital and was Health Insurance Portability and Accountability Act-compliant.

### 2.2. Data Extraction

Demographic and clinical data were extracted from electronic medical records retrospectively. The following variables were acquired: age, sex, race, body mass index, smoking status, alcohol use, comorbidities (hypertension, hyperlipidemia, diabetes mellitus, heart disease (coronary artery disease, heart failure), atrial fibrillation), past medical history (history of malignancy, prior cerebrovascular accident), vitals on admission (heart rate, systolic blood pressure, diastolic blood pressure, respiratory rate), anticoagulant use, admission NIHSS score, intravenous (IV) tissue plasminogen activator (tPA) treatment, time from symptom onset to CT acquisition, and modified Rankin Scale (mRS) score at 90 days. The mRS score at 90 days was used to assess the level of disability in stroke patients on an ordinal scale ranging from 0 (no symptoms) to 6 (death). Based on the recent trial from Yoshimura et al. (RESCUE-Japan Limit), the clinical outcome was dichotomized into favorable functional outcome (mRS 0–3) or unfavorable (mRS 4–6) [17].

All patients had peripheral venous blood samples drawn at the emergency department per our local stroke care standard protocol. All blood samples were collected and processed the same way and tested at the same clinical laboratory. The following baseline parameters were extracted retrospectively: glucose, sodium, potassium, calcium, blood urea nitrogen-to-creatinine ratio (BUN/Cr), hemoglobin, hematocrit, mean corpuscular volume (MCV), platelet count (PC), mean platelet volume (MPV), neutrophil count, and neutrophil-to-platelet ratio (NPR).

Radiologic variables were gathered from imaging and procedure notes. The Alberta Stroke Program Early CT Score (ASPECTS), occluded vessel and its segment, laterality of the occlusion, and presence and type of hemorrhagic transformation were collected and verified by a board-certified neuroradiologist (VSY, 6 years of experience). The ASPECTS was used to assess early ischemic changes in pretreatment CT studies [18]. Based on European Cooperative Acute Stroke Study-II criteria, hemorrhagic transformation was classified as hemorrhagic infarction types 1 and 2 or parenchymal hematoma types 1 and 2 [19]. If IV tPA was used prior to MT, the time from the last known normal to the needle time in minutes (NTN) was collected.

Thrombectomy was performed by one of four experienced interventional neuroradiologists or endovascular neurosurgeons using any FDA-approved thrombectomy device at the discretion of the neurointerventionalist. Utilized techniques included direct aspiration thrombectomy using 6-French and 5-French distal aspiration catheters, retrievable stent mechanical thrombectomy, or combination distal aspiration and retrievable stent mechanical thrombectomy with or without the use of a balloon guide catheter in accordance with current technical standards [20]. Guideline selection criteria were followed in accordance with center protocol [21], and in the majority of cases, CT perfusion data were acquired and analyzed during initial patient evaluation. IV tPA was administered prior to femoral puncture if indicated. For patients with stenosis or occlusion of the cervical ICA due to atherosclerosis, carotid angioplasty with or without stenting was permitted after multidisciplinary consideration. General endotracheal anesthesia with continuous blood pressure monitoring using a pressure transducing arterial sheath was utilized in most cases. Standard medical therapy, based on current American Heart Association guidelines, was administered to all patients prior to and after mechanical thrombectomy [21].

Procedure data were recorded at the time of intervention using a standard reporting format. Abstracted data included: (1) the time from last known normal to groin puncture in minutes; (2) the time from groin puncture to recanalization in minutes; (3) the reperfusion grade as assessed by the treating interventionalist at the conclusion of the procedure using the mTICI score; and (4) the number of passes during the thrombectomy procedure.

### 2.3. Statistical Analysis

We calculated frequencies for categorical variables, means with standard deviations for normally distributed continuous variables, and medians with interquartile ranges for non-normally distributed continuous variables. We performed an independent *t*-test for normally distributed continuous variables with equal variances and a Mann–Whitney U test for non-normally distributed continuous variables to identify the relationship between continuous variables and mRS groups. We used Fisher’s exact test for the categorical variables. Variables with a *p*-value less than 0.2 were included in the multivariable logistic regression analysis. Finally, predictive models were built using variables with a *p*-value less than 0.05 in the multivariable logistic regression analysis. The models were built separately for each variable and with all variables combined. Receiver operating characteristic (ROC) curves were estimated to assess the model’s predictive ability. A *p*-value of <0.05 was considered statistically significant. All statistical analyses were performed in R 4.1.3 (R Foundation for Statistical Computing, Vienna, Austria) [22] with RStudio 2022.02.1 + 461 (RStudio: Integrated Development for R. RStudio, PBC, Boston, MA, USA) [23].

## 3. Results

A total of 53 patients treated with MT with an mTICI score of 2b or higher were included in the study. Patients with missing data were excluded. There were 26 patients in the favorable outcome group and 27 in the unfavorable outcome group. Baseline clinical, laboratory, and radiologic characteristics of the patient population are presented in Table 1. Age, BUN/Cr, PC, calcium, NPR, history of diabetes mellitus, alcohol use, and time from last known normal to groin puncture in minutes were discovered to have *p*-values of less than 0.2 in the univariate analyses.

**Figure 1:**
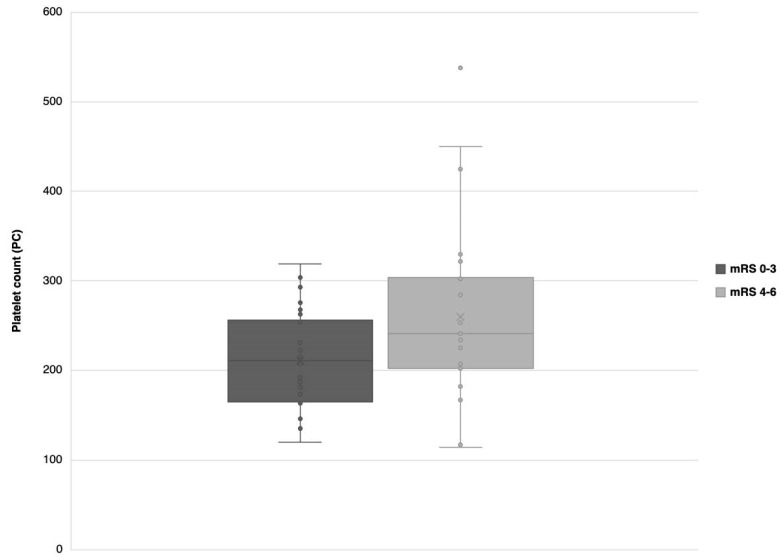
Box and whisker plots for PC values in patients with favorable and unfavorable outcomes. Abbreviations: modified Rankin Scale (mRS), platelet count (PC).

The multivariate logistic regression analysis revealed age and PC as predictors of unfavorable outcomes (Table 2). These two were used to create the three prediction models (Table 3). PC values for patients with favorable and unfavorable outcomes were described using box and whisker plots (Figure 1).

**Figure 2:**
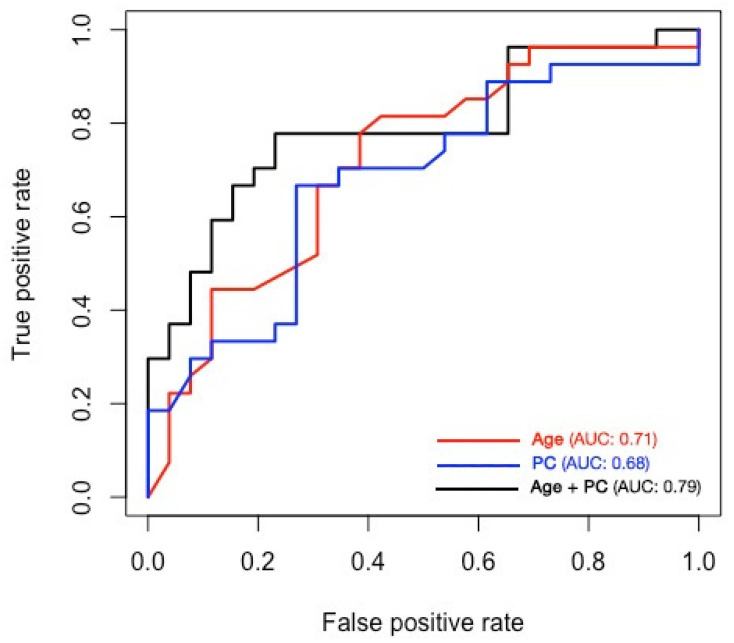
The areas under the ROC curve of models 1, 2, and 3. Abbreviations: area under the curve (AUC), platelet count (PC), receiver operating characteristic (ROC).

Model 1 was based on the age only: logit (mRS 4–6) = −3.231 + 0.047 × age. Setting the probability threshold to 0.49 yielded a sensitivity of 77.8% and a specificity of 61.5%. Model 2 was based on the PC only: logit (mRS 4–6) = −2.169 + 0.01 × PC. Setting the probability threshold to 0.51 yielded a sensitivity of 66.7% and a specificity of 73.1%. Model 3 was based on both the age and the PC: logit (mRS 4–6) = −6.484 + 0.055 × age + 0.012 × PC. Setting the probability threshold to 0.48 yielded a sensitivity of 77.8% and a specificity of 76.9%. The areas under the ROC curve (AUCs) of models 1, 2, and 3 were 0.71, 0.68, and 0.79, respectively (Figure 2).

In patients with treated tPA prior to MT, NTN was recorded only in 24 patients, 10 of whom had unfavorable outcomes and 14 of whom had favorable outcomes. As a result, NPN values were not used in the logistic regression analyses. The median NTN was 117 (94.5) in unfavorable patients and 150 (121) in favorable patients.

## 4. Discussion

Our study found an independent association between a high admission PC and unfavorable clinical outcomes presenting with AIS secondary to LVO with an NIHSS score ≥ 21 in patients who were subsequently successfully treated with MT. Furthermore, we found that older age was an independent risk factor for an unfavorable clinical outcome. This finding is in line with other studies on endovascular therapy in stroke patients [24,25]. We also developed a prediction model based on the results of the multivariate analysis that accurately predicts the unfavorable clinical outcome using age and PC. To the best of our knowledge, this is the first study to investigate these prognostic factors within this very specific subgroup. Therefore, our novel findings should be considered preliminary, requiring additional studies.

Our study showed that PC values were useful in predicting unfavorable outcomes. When PC values were used alone, the area under the ROC curve was 0.68; when PC values were combined with age, it was 0.79. Platelets have been shown to play an important role in the pathogenesis of atherothrombosis and AIS [26]. They contribute to AIS pathophysiology by facilitating the formation of thromboemboli [27]. Our study included an elderly population with an average age of 67.89 (±18.19). In addition to demonstrating a positive association between platelet aggregation and PC values, Karolczak et al. also showed that in older adults, PC values also serve as a predictor of platelet reactivity [28]. Moreover, Viallard et al. showed a positive correlation between PC values and the plasma concentrations of soluble levels of CD40 ligand, which promotes prothrombotic states [29]. Furthermore, through interactions with leukocytes, the vessel wall, and by deposition of chemoattractants on the vessel wall, platelets participate in the inflammatory process underlying large vessel disease [30]. This inflammatory response that is platelet-dependent may contribute to tissue injury in AIS patients [30,31]. In addition, a potent chemoattractant of inflammatory cells called platelet-derived RANTES accumulates on the endothelium and contributes to the inflammation and injury of the tissue [32,33]. We believe these valuable findings support the significance of the admission PC association with unfavorable outcomes discovered in our study. However, Chen et al. and Sotero et al. showed no association between PC and outcome in AIS patients [34,35]. They both included patients with any admission NIHSS score. Furthermore, while Chen et al. included patients treated with MT regardless of recanalization success, Sotero et al. included patients with IV thrombolysis regardless of recanalization success. Differences in patient cohorts could explain the differences in these studies’ findings. In addition, several studies have found that an increased MPV value is associated with unfavorable outcomes in stroke patients [24,35,36]. MPV reflects platelet functional changes and activation based on average platelet size [37]. One of the probable explanations for the association between an elevated MPV and treatment outcomes in stroke patients is that patients with higher MPV have greater platelet activation, leading to lower recanalization rates and worse results [12,38,39]. In contrast to those studies, our sample only included patients who were successfully recanalized with MT, perhaps rendering the possible explanation inapplicable; thus, we did not find any significant association between MPV values and unfavorable outcomes. Furthermore, unlike the aforementioned studies reporting an association between unfavorable outcomes and MPV in stroke patients [24,35,36], our cohort only included patients with an admission NIHSS score ≥ 21, implying that MPV may have been insignificant in our subgroup. The relationship between increased MPV and prognosis in patients with AIS is still debatable, so larger-scale studies are required [40].

In our study representing patients with anterior circulation LVO and severe stroke symptoms successfully treated with MT, the admission NIHSS score was not associated with mRS at 90 days. Prior studies, in contrast, have reliably shown that the NIHSS score on admission is a predictive factor of outcomes after MT [41]. By design, our mean admission NIHSS score of 21 is considerably higher than previous studies. While the NIHSS score is a reproducible method for assessing stroke severity among properly trained examiners [42], the score does have limitations and is not intended to represent a comprehensive assessment of a patient’s neurological status [43]. The ability of the score to reliably discriminate between patients with severe and very severe stroke symptoms, for example, has not been evaluated. Thus, in this population with high NIHSS scores, it is possible that a distinction between patients with severe and very severe symptoms was not achieved, and a difference in outcome could not be observed. It is also possible that due to the methodological design selecting for high NIHSS scores, the presence of LVO, and meeting qualifying clinical and imaging criteria for MT, that a very homogeneous study population was selected regarding stroke severity, which could also explain a lack of observed effect.

Chang et al. demonstrated that using IV tPA prior to MT improves functional outcomes [44]. However, their sample also included patients who were unable to be successfully revascularized following MT. Prior use of tPA may have no bearing on patient functional outcomes in the case of successful revascularization with MT in severe stroke patients because the patient is revascularized regardless, as there was no statistical difference between those treated with IV tPA in our favorable and unfavorable outcome subgroups in our study.

Time from symptom onset to groin puncture, time from groin puncture to recanalization, and mTICI = 2b have previously been documented to be negative prognostic factors among patients successfully treated with MT [6,7,8,9,45,46]. Significant associations with these variables and outcome, however, were not observed in our study. Due to the methodological choice to focus on successfully treated MT patients, some of these effects could have been attenuated. Additionally, our study population represented both early window (presenting within 6 h) and late window (presenting between 6 and 24 h) patients, and the determinants of outcomes among these groups, particularly with regard to time, have been shown to be different [46]. These differences could have also been affected by the use of advanced imaging in the majority of cases in all time windows. Finally, some established prognostic factors such as procedure time did in fact show trends consistent with the prior literature, although observed differences did not reach statistical significance.

Although we recognize that the relatively small number of patients in our study is a limitation, it should be noted that our research focuses on a particular but important subgroup and serves as a crucial preliminary study. According to one study, only 5.61% of all stroke patients and 4.49% of ischemic stroke patients had an admission NIHSS score of 21 or higher in 2013 in Israel [47]. Another study found that higher admission NIHSS scores are associated with a worse outcome [14]. It is important to note that since our study had a small number of patients from a single center, it should be considered a pilot, preliminary study and evaluated as such.

The inherent limitations of observational studies served as the main limitation for this study. The study’s focus on a very specific clinical group only including severe strokes with LVOs resulted in a small sample size, which was the second limitation. Furthermore, since PC is a non-brain-specific indicator and no known baseline is predefined for the different neurological disorders, these results should be evaluated in a large cohort to avoid any false generalization. Therefore, additional large-scale, multicenter studies are needed to determine the role of PC values in the prognosis of this specific group. Moreover, while we excluded patients with accompanying intracranial hemorrhage, we did not account for previous, potentially confounding brain injuries. Furthermore, residual cholesterol and inflammatory risk, as indicated by baseline low-density lipoprotein cholesterol and high-sensitivity C-reactive protein levels, have been linked to the risk of a poor functional outcome [48]. Although inflammation plays a significant role in the pathogenesis and prognosis of AIS, we did not consider any inflammatory markers other than neutrophil count. Further research would be needed with the consideration of brain-specific markers, more pro- and anti-inflammatory mediators, and resident cells in the brain [49]. Finally, the study did not consider antiplatelet therapy and subtypes of ischemic stroke.

## 5. Conclusions

In conclusion, this is the first study to reveal that elevated PC is an independent predictor of unfavorable outcomes in patients presenting with severe AIS, defined as an admission NIHSS score ≥ 21, with anterior circulation LVO, who underwent successful MT. Baseline PC may be a valuable indicator for risk stratification in this group as an inexpensive marker. The platelet-dependent inflammatory response in stroke patients may be important for prognosis and should be studied further. Since this is a preliminary study, large-scale studies are needed to validate our findings.

## Figures and Tables

**Table 1:** Baseline clinical, laboratory, and radiologic characteristics of the patient population.

	Total	Favorable Outcome (mRS Score 0–3, n = 26)	Unfavorable Outcome (mRS Score 4–6, n = 27)	*p*-Value
Age	67.89 ± 18.1	61.04 ± 17.86	74.48 ± 16.02	0.006 *†
Sex				1.000
Female	30 (56.6%)	15 (57.7%)	15 (55.6%)	
Male	23 (43.4%)	11 (42.3%)	12 (44.4%)	
Race				0.586
White	27 (50.9%)	14 (53.8%)	13 (48.1%)	
Black/African American	25 (47.2%)	11 (42.3%)	14 (51.9%)	
Other	1 (1.9%)	1 (3.8%)	0	
BMI	26.42 (9.7)	26.51 (9.1)	26.42 (9.4)	0.965
Smoking status				0.587
No	27 (50.9%)	12 (46.2%)	15 (55.6%)	
Yes	26 (49.1%)	14 (53.8%)	12 (44.4%)	
Alcohol use				0.148 †
No	36 (67.9%)	15 (57.7%)	21 (77.8%)	
Yes	17 (32.1%)	11 (42.3%)	6 (22.2%)	
Hypertension				0.526
No	12 (22.6%)	7 (26.9%)	5 (18.5%)	
Yes	41 (77.4%)	19 (73.1%)	22 (81.5%)	
Hyperlipidemia				0.779
No	33 (62.3%)	17 (65.4%)	16 (59.3%)	
Yes	20 (37.7%)	9 (34.6%)	11 (40.7%)	
Diabetes mellitus				0.119 †
No	39 (73.6%)	22 (84.6%)	17 (63%)	
Yes	14 (26.4%)	4 (15.4%)	10 (37%)	
Heart disease				0.264
No	32 (60.4%)	18 (69.2%)	18 (66.7%)	
Yes	21 (39.6%)	8 (30.8%)	13 (48.1%)	
Atrial fibrillation				1.000
No	35 (66%)	17 (65.4%)	18 (66.7%)	
Yes	18 (34%)	9 (34.6%)	9 (33.3%)	
History of malignancy				0.467
No	45 (84.9%)	21 (80.8%)	24 (88.9%)	
Yes	8 (15.1%)	5 (19.2%)	3 (11.1%)	
Prior cerebrovascular accident				1.000
No	38 (71.7%)	19 (73.1%)	19 (70.4%)	
Yes	15 (28.3%)	7 (26.9%)	8 (29.6%)	
Heart rate	85.62 ± 19.8	85.35 ± 22.31	85.89 ± 17.48	0.922
Systolic blood pressure	143 (29)	142.5 (24)	144 (32.5)	0.810
Diastolic blood pressure	83 (24)	83.5 (20.5)	82 (23)	0.423
Respiratory rate	19 (6)	18 (5.75)	19 (4.5)	0.781
Anticoagulant use				1.000
No	31 (58.5%)	15 (57.7%)	16 (59.3%)	
Yes	22 (41.5%)	11 (42.3%)	11 (40.7%)	
Admission NIHSS	25 (4)	24.5 (3)	25 (5)	0.687
IV tPA treatment				0.412
No	30 (56.6%)	13 (50%)	17 (63%)	
Yes	23 (43.4%)	13 (50%)	10 (37%)	
Time from symptom onset to CT in minutes	150 (187)	132 (126.8)	186 (234.5)	0.838
Glucose	120 (41)	118 (33)	122 (43)	0.831
Sodium	139.28 ± 3.98	139.35 ± 3.39	139.22 ± 4.54	0.911
Potassium	4.08 ± 0.55	4 ± 0.52	4.16 ± 0.58	0.309
Calcium	8.8 (1.1)	9.05 (1.3)	8.7 (1)	0.076 †
BUN:Creatinine ratio	15.4 (10)	13.5 (7.8)	21 (11.8)	0.020 *†
Hemoglobin	12.6 (2)	12.85 (1.7)	12.6 (2.1)	0.563
Hematocrit	38.8 (6.3)	38.85 (4.8)	38.8 (6.6)	0.957
Mean corpuscular volume	90.6 (10.7)	90.8 (10.2)	89.8 (8.2)	0.298
Platelet count	231 (86)	211 (81.3)	241 (98.5)	0.026 *†
Mean platelet volume	10.59 ± 0.97	10.71 ± 1	10.48 ± 0.96	0.395
Neutrophil count	6888 (6206)	6705 (5674)	6919 (6529)	0.531
Neutrophil:Platelet ratio	33.1 (2.8)	36.35 (21.1)	30.75 (19.6)	0.115 †
Baseline NCCT ASPECTS	9 (3)	9 (2.8)	9 (2.5)	0.812
Occlusion site on CT				0.624
Distal intracranial ICA only	8 (15.1%)	2 (7.7%)	6 (22.2%)	
M1 only	33 (61.1%)	18 (69.2%)	15 (55.6%)	
M1 and M2	5 (9.4%)	2 (7.7%)	3 (11.1%)	
ICA and M1	3 (5.7%)	2 (7.7%)	1 (3.7%)	
M2 only	4 (7.5%)	2 (7.7%)	2 (7.4%)	
Occlusion laterality				0.372
Left	37 (69.8%)	20 (76.9%)	17 (63%)	
Right	16 (30.2%)	6 (23.1%)	10 (37%)	
Hemorrhagic transformation on post-procedural follow up within 48 h				0.704
No	45 (84.9%)	23 (88.5%)	22 (81.5%)	
Yes	8 (15.1%)	3 (11.5%)	5 (18.5%)	
Time from last known normal to groin puncture in minutes	208 (141)	246 (165.5)	190 (131)	0.168 †
Time from groin puncture to recanalization in minutes	35 (42)	31.5 (42)	42 (42)	0.444
Number of passes in thrombectomy	1 (2)	1 (2)	1 (1.5)	0.861
mTICI score category				0.351
2b	21 (39.6%)	12 (46.2%)	9 (33.3%)	
2c	8 (15.1%)	2 (7.7%)	6 (22.2%)	
3	24 (45.3%)	12 (46.2%)	12 (44.4%)	

Data are presented as the mean ± standard deviation, n (%), or median (interquartile range). * Significant difference (*p* < 0.05) upon statistical testing. † Variables with a *p*-value less than 0.2. Abbreviations: modified Rankin Scale (mRS), body mass index (BMI), National Institutes of Health Stroke Scale (NIHSS), intravenous (IV), tissue plasminogen activator (tPA), computed tomography (CT), blood urea nitrogen (BUN), non-contrast CT (NCCT), Alberta Stroke Program Early CT Score (ASPECTS), internal carotid artery (ICA), modified treatment in cerebral ischemia (mTICI).

**Table 2:** Results of the multivariate logistic regression.

Variable	OR (95% CI)	*p*-Value
Age	1.051 (1.015–1.121)	0.025 ‡
Platelet count	1.014 (1.003–1.029)	0.031 ‡
Calcium	0.343 (0.074–1.005)	0.115
BUN:Creatinine ratio	1.077 (0.976–1.206)	0.157
Neutrophil:platelet ratio	0.940 (0.976–1.022)	0.342
Alcohol use (yes)	0.423 (0.063–2.446)	0.344
Time from last known normal to groin puncture in minutes	1.001 (0.998–1.004)	0.572
Diabetes mellitus (yes)	1.400 (0.226–9.342)	0.716

‡ Significant difference (*p* < 0.05) upon statistical testing. Abbreviations: odds ratio (OR), confidence interval (CI), blood urea nitrogen (BUN).

**Table 3:** Results of the predictive models.

Variable	B-Coefficient (95% CI)	OR (95% CI)	*p*-Value
Model 1			
Intercept	−3.231 [(−6.141)–(−0.832)]	-	-
Age	0.048 (0.014–0.089)	1.049 (1.014–1.092)	0.011
Model 2			
Intercept	−2.169 [(−4.513)–(−0.231)]	-	-
Platelet Count	0.009 (0.002–0.019)	1.010 (1.002–1.020)	0.038
Model 3			
Intercept	−6.484 [(−11.385)–(−2.764)]	-	-
Age	0.055 (0.018–0.102)	1.057 (1.018–1.107)	0.009
Platelet Count	0.012 (0.003–0.024)	1.012 (1.003–1.024)	0.029

Abbreviatons: confidence interval (CI), odds ratio (OR).

## Data Availability

The data presented in this study are available on request from the corresponding author. The data are not publicly available due to ethical restrictions and legal constraints.
